# Sodium Patterns and Their Variables in a Cohort of ELBW Infants in the First 10 Days of Life

**DOI:** 10.3390/children12030337

**Published:** 2025-03-07

**Authors:** Stijn van Sas, Myrna Pace, Thomas Salaets, Annouschka Laenen, Anke Raaijmakers, Karel Allegaert

**Affiliations:** 1 Faculty of Medicine, KU Leuven, 3000 Leuven, Belgium; stijn.vansas@student.kuleuven.be (S.v.S.); myrna.pace@student.kuleuven.be (M.P.); 2 Pediatric Cardiology, University Hospitals, 3000 Leuven, Belgium; thomas.salaets1@uzleuven.be; 3 Leuven Biostatistics and Statistical Bioinformatics Center (L-BioStat), KU Leuven, 3000 Leuven, Belgium; annouschka.laenen@kuleuven.be; 4 Department of Paediatric Nephrology, Sydney Children’s Hospital, Randwick, NSW 2031, Australia; anke.raaijmakers@health.nsw.gov.au; 5 School of Women’s and Children’s Health, University of New South Wales, Kensington, NSW 2031, Australia; 6 Department of Development and Regeneration, KU Leuven, 3000 Leuven, Belgium; 7 Department of Pharmaceutical and Pharmacological Sciences, KU Leuven, 3000 Leuven, Belgium; 8 Department of Hospital Pharmacy, Erasmus MC, 3015 GD Rotterdam, The Netherlands

**Keywords:** extremely low birth weight, hyponatremia, hypernatremia, sodium

## Abstract

**Background**: Sodium regulation is critical in extremely low-birth-weight (ELBW, <1000 g) infants. In a recent systematic review, a sodium pattern over postnatal age and its variables (care factors, fluid regimens, and maturational factors) has been summarized. However, this systematic review also illustrated the shortages and limitations of reported cohorts, and the need to report on additional datasets. This study therefore aims to describe the postnatal sodium patterns and their variables in a cohort of ELBW neonates in the first 10 days of postnatal life. **Methods**: Data on 1704 serum sodium observations in the first 10 days of life from 211 ELBW infants hospitalized in a single neonatal intensive care unit were available to explore associations between serum sodium and perinatal variables. Multivariate linear models with sodium as a response variable and postnatal day as a factor were hereby applied. Baseline and treatment characteristics were included as variables, applying an unstructured covariance matrix to account for the longitudinal data. **Results**: Gestational age, birth weight, and length showed variable correlations with serum sodium concentrations over postnatal age. Interestingly, the analysis of sodium patterns in this ELBW cohort also revealed significant associations between prenatal betamethasone use, delivery mode, ibuprofen, or the use of inotropes and the postnatal serum sodium concentrations patterns. Multivariate analyses confirmed that gestational age and birth weight independently impacted sodium concentration patterns, and that ibuprofen use remained a significant variable after adjusting for these variables. **Conclusions**: Gestational age and birth weight complexities emphasize the need for nuanced understanding and standardized methodologies. Sodium patterns in the current ELBW cohort provide support for previously published sodium reference patterns in this population. New variables associated with sodium levels include ibuprofen administration and the use of inotropic agents.

## 1. Introduction

While significant improvements in survival and outcome occurred over the last decades, extremely low-birth-weight (ELBW, <1000 g) infants still have to confront short- as well as long-term outcome variables [1,2]. Sodium dysregulation, particularly in the first 10 days of life, poses a significant concern as a modifiable indicator of short- and long-term outcomes. Unfortunately, aspects of patterns, reference values, or variables of sodium remain rather poorly reported in this specific ELBW population [3].

In a recent systematic review on this topic, patterns on sodium changes over postnatal age in ELBW cases were summarized [4]. Besides care-related variables (incubator humidity and temperature settings, occlusive skin care, or fluid regimens), these patterns are mainly related to maturational factors of the ELBW infant (age-related aspects, like postnatal or gestational age, or being either appropriate or small for gestational age, AGA, or SGA, respectively). However, only eight studies (observational studies, n = 5; case control studies, n = 2; and one randomized controlled trial) were retained [4]. Consequently, the extensive heterogeneity among the studies retained in this review called for further reflections and interpretation of the pooled data, while additional validation studies are needed to better document normative values and to improve electrolyte (like sodium) or fluid supplementation practices and protocols. Because of the specific characteristics of the sodium values as reported in the individual papers, we also failed to provide data on mean sodium values over postnatal age [4]. This makes studies on, e.g., associations between hyper- or hyponatremia and outcome variables even more difficult to interpret.

Since the recently published systematic review illustrated the shortage of reported cohorts, hereby confirmed as being relevant to the new datasets, this study aims to describe the early postnatal sodium patterns and their variables in a large cohort of ELBW neonates.

## 2. Materials and Methods

### 2.1. Clinical Characterstics and Fluid Administration Strategy

This study utilized previously collected longitudinal serum sodium concentrations of neonates (neonatal intensive care unit (NICU), University Hospitals Gasthuisberg Leuven, July 2007–August 2011), pooled with another dataset (June 2015–March 2017) from the same unit. All ELBW infants who had sodium serum observations collected within the first 10 days were included [5]. We defined day 1 as the first 24 h of life [6]. In addition, clinical data [birth weight, length, gestational age, SGA or AGA, sex, neonatal death, prenatal lung maturation, type of delivery (caesarean/vaginal), ibuprofen prescription, and exposure to inotropes] were extracted from medical records as previously described [5]. Ethics approval (S63405, KU Leuven, Belgium) has been provided to extract these data and perform the analysis [5].

All ELBW neonates were cared for in closed incubators, with temperature setting to sustain normothermia and with humidity settings at 50–80%. The fluid and sodium management in this cohort was standardized based on the Leuven protocol, with an increment in fluid volume over postnatal age (60 mL/kg, based on 40 mL/kg/24 h glucose 10% + 20 mL/kg plasma on day 1; day of birth—day of birth, 80 mL/kg/24 h on day 2; 100 mL/kg on day 3; 120 mL/kg/24 h on day 4; and 150–180 mL/kg/24 h for day 5 onwards). Sodium (2.5 mmol/kg/day) was commonly administrated as part of parenteral nutrition from day 4 onwards [7].

### 2.2. Statistical Analysis

Statistical analysis explored the patterns and trends in sodium levels in the first 10 days of postnatal life, and how these patterns were associated with perinatal and neonatal variables. The variables included prenatal betamethasone lung maturation treatment, mode of delivery, SGA status, gestational age, sex, birth weight and length, ibuprofen treatment, and inotropic agents. We applied multivariate linear models with sodium as the response variable and postnatal day as the factor. Baseline or treatment characteristics were included as variables, with an unstructured covariance matrix to account for longitudinal data.

We tested interactions between day and each characteristic, presenting mean differences with 95% confidence intervals (CIs) per day if significant, or overall effects if not. If applicable, non-linear effects of continuous variables were estimated using log-transformation. Results were presented as slopes with 95% Cis, applying SAS software (SAS System for Windows, version 9.4) [8].

## 3. Results

### 3.1. Clinical Characteristics of the ELBW Cohort

Data were collected on 211 ELBW neonates, with population characteristics, as well as the prescription of betamethasone (prenatal lung maturation), ibuprofen, and inotropic agents, as summarized in Table 1.

**Figure 1:**
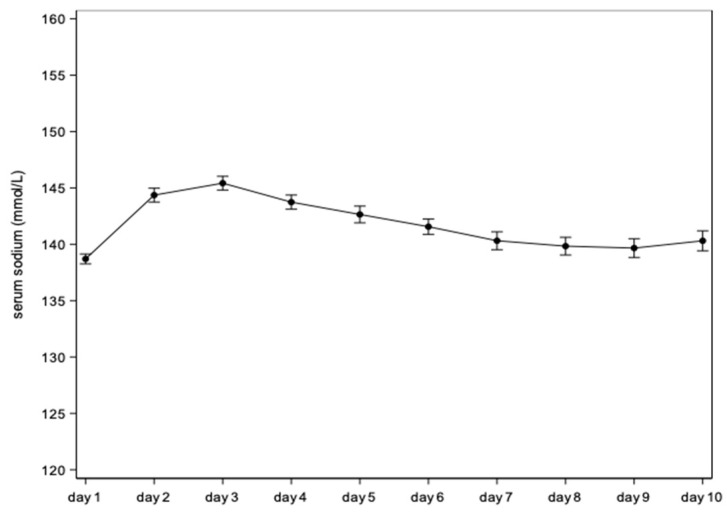
Mean daily serum sodium concentrations in extremely low-birth-weight (ELBW) infants between day 1 and day 10 (95% confidence intervals).

The study measured 1704 serum sodium concentrations in the cohort of ELBW newborns in their first 10 days of postnatal life. Figure 1 illustrates the variation of serum sodium concentration over time. Serum sodium was significantly higher after day 1, with a peak serum sodium concentration on day 3 at 145.4 mmol/L, followed by a subsequent blunted decrease until day 9.

Table 2 displays the daily mean (95% confidence interval) of sodium concentrations starting from the day of birth (referred to as day 1). The mean difference versus day 1 hereby quantifies the trends illustrated in Figure 1.

### 3.2. Perinatal Variables

**Figure 2:**
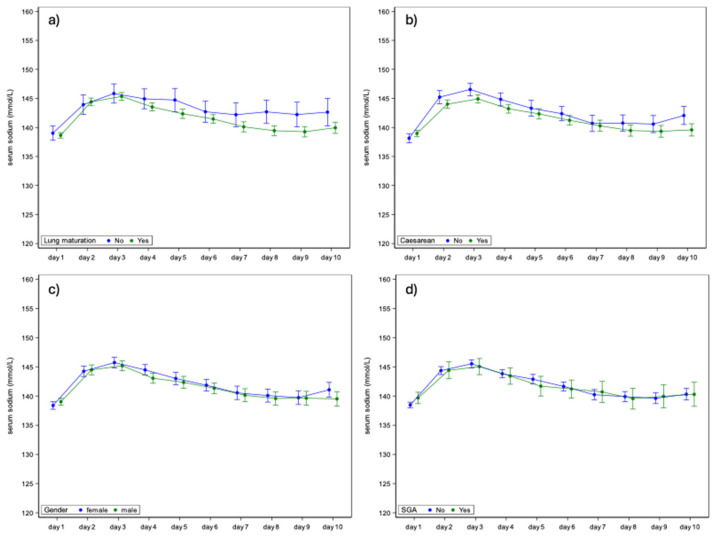
(**a**) Serum sodium concentrations of ELBW infants with and without prenatal betamethasone lung maturation treatment in the first 10 days. (**b**) Serum sodium concentrations of ELBW infants born by caesarean section vs. vaginal delivery. (**c**) Serum sodium concentrations female vs. male ELBW infants. (**d**) Serum sodium concentrations of SGA and AGA ELBW. (Extremely low birth weight, ELBW; small for gestational age, SGA; and appropriate for gestational age, AGA).

We evaluated the associations between prenatal betamethasone lung maturation administration and sodium serum concentration in ELBW infants over postnatal age (day 1 until day 10) but found no significant correlations. The mean serum sodium concentration did not differ significantly between infants with or without lung maturation treatment (Figure 2a). Additionally, we studied the impact of delivery mode (caesarean vs. vaginal) on serum sodium concentrations (Table S1). While some differences were noted on specific days, overall, no consistent significant associations were found (Figure 2b).

### 3.3. Neonatal Variables

Associations between neonatal variables such as sex (Figure 2c), SGA status (Figure 2d), and serum sodium concentrations were evaluated. No significant associations were observed. The serum sodium concentrations between male and female ELBW infants, or between SGA and AGA infants, were not significantly different.

**Figure 3:**
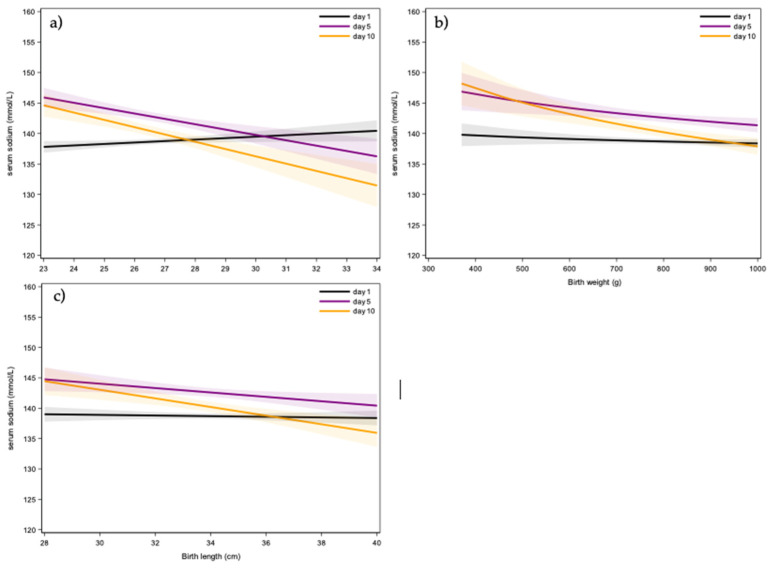
The graphs each show the slope and 95% confidence interval (CI) of the associations between gestational age (**a**), birth weight (**b**), and birth length (**c**), respectively, and serum sodium concentrations for each day. The slope represents the change in serum sodium for a 1-week higher gestational age, for a 2-fold higher birth weight, and for a 2-fold higher birth length, respectively.

We also assessed the impact of gestational age (Figure 3a, Table S2), birth weight (Figure 3b, Table S3), and birth length (Figure 3c) on serum sodium concentration. The associations for these different variables varied across the postnatal time interval explored (day 1–10). Specifically, gestational age showed a positive correlation on day 1 and a negative correlation on the subsequent days (Figure 3a). Birth weight and birth length were negatively associated with serum sodium concentrations from day 3 onward (Figure 3b,c, Tables S9 and S10).

**Figure 4:**
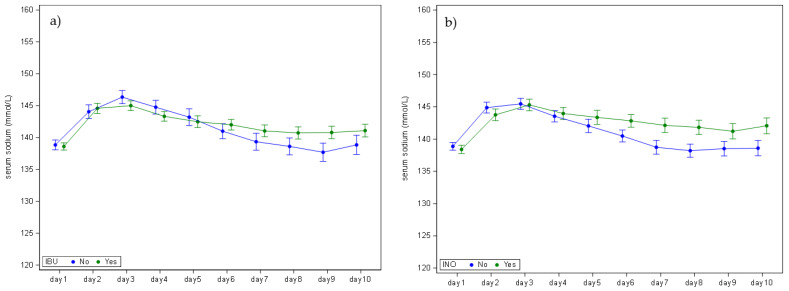
(**a**) Comparing sodium values in cases either exposed or not exposed to ibuprofen, corrected for gestational age. (**b**) Comparing sodium values in cases either exposed or not exposed to ionotropic agents.

We compared serum sodium concentrations between infants who were exposed to ibuprofen and those who were not (Figure 4a, Table S4), as well as between those exposed to inotropic agents and those who were not exposed (Figure 4b, Table S5). Both ibuprofen and inotropic agent exposure showed significant day-to-group interactions, with differences in sodium concentrations observed from day 6 onwards (Tables S4 and S5).

Multivariate analyses confirmed that gestational age and birth weight independently impacted sodium levels, and that ibuprofen use remained a significant variable after adjusting for these variables (Table S6).

## 4. Discussion

Sodium homeostasis in neonates is very important, most prominent in the most immature ELBW subpopulation. This study reports on an analysis of sodium patterns and the variables associated of these sodium values in a cohort of 211 ELBW’s from a single neonatal intensive care unit.

When considering the overall pattern over postnatal life (Figure 1, Table 2), we noticed the expected increase in sodium values over the first days of life, followed by a subsequent blunted decline to attain sodium values similar to those at birth from the end of the first week of life onwards. This pattern largely confirms the pattern recently described in the systematic review [4], assuming that the initial increase in sodium levels is enhanced by a physiological fluid shift in the very first days of life (more pronounced water losses), to attain a peak on day 3, followed by a gradual decrease [1,2,3,4]. In this way, the currently reported sodium reference values validate those previously described findings [4].

Since ELBW infants display prolonged water losses, sodium and fluid supplementation after initial weight loss are necessary [1,9,10]. The targeted outcome hereby lies in titrating the need for fluids to avoid hypernatremia, as well as dehydration or hyperbilirubinemia, while at the very same time mitigating the potential complications of excessive fluid administration like patent ductus arteriosus, or bronchopulmonary dysplasia. In the best case scenario, appropriate fluid supplementation should allow sufficient contraction of the extracellular space while still avoiding excessive fluid loss to avoid early hypernatremia and providing sufficient sodium supplement to facilitate postnatal growth [9,10]. Costarino et al. [11] (bronchopulmonary dysplasia) and Eibensteiner et al. [12] (high-grade intraventricular hemorrhage, necrotizing enterocolitis, and mortality) observed that sodium intake restriction resulted in better outcomes [11,12].

When reflecting on the variables associated with sodium patterns in the first 10 days of postnatal age, gestational age’s association with sodium values varied across the first 10 days in the current analysis. Higher gestational age was correlated with higher sodium values on day 1, while days 3–10 showed a correlation with lower sodium values. It is important to stress that newborns who were born at 34 weeks of gestational age yet <1 kg were severely growth-restricted. Correspondingly, Boubred et al. reported that there was a non-significant trend to lower sodium serum values in SGA newborns, compared to AGA newborns [13]. The sodium decreases associated with a decreasing gestational age observed in the research of Strizke et al. align with the finding from our cohort on day 1 of life [14].

Similarly, the effect of birth weight on sodium serum values also varied, with lower sodium values associated with higher birth weight, on all days analyzed, except for days 1 and 2. Higher birth weight was associated with lower average sodium values. Monnikendam et al. also reported a link between moderate and severe hypernatremia and lower gestational age, or birth weight [3].

Besides largely confirming the relevance of these maturational differences, additional and new variables were identified. Infants who received ibuprofen had higher sodium levels than those who were not exposed. This was somewhat unexpected as non-steroidal anti-inflammatory drugs (NSAIDs) inhibit prostaglandin synthesis, potentially causing a range of disturbances in acid-base or electrolyte balance and inducing fluid retention. These disturbances can include sodium retention and a decrease in renal function, which can further reduce renal free water clearance and lead to hyponatremia [15]. Additionally, ibuprofen can also impact renal function directly by causing tubular toxicity, leading to a decrease in the concentrating ability of the kidneys. A likely explanation for the hypernatremia observed in this cohort is that the infants who were administered ibuprofen also had fluid restriction as a part of their treatment [15].

Sodium values were significantly higher for infants who were exposed to ionotropic agents compared to their counterparts. Inotropes are commonly used in NICUs to treat hypotension and poor perfusion. However, their impact on serum sodium levels in neonates is not well-studied, and the evidence is limited [16]. One possible explanation could be related to the physiological effects of inotropes. They can increase blood pressure and improve organ perfusion, which might influence renal function and subsequently affect electrolyte balance, including sodium levels [16]. Another variable could be the fluid management in neonates who are on inotropes. These neonates likely receive additional sodium containing fluid boluses compared to those not on inotropes, which could influence their serum sodium levels. However, it is important to note that this is a complex issue, and other variables could also be involved. More research is warranted to further explore the relationship between inotrope prescription use, and sodium serum levels in neonates.

Interestingly, infants born by vaginal delivery in the current cohort exhibited higher sodium values on days 3, 4, and 10 compared to those born by caesarean section. To our knowledge, this association has not yet been observed, and we can only speculate on the underlying mechanisms, perhaps related to peripartal fluid shifts. Stritzke et al. looked at the impact of caesarean delivery on cord blood electrolytes, but no significant association was observed after correcting for gestational age [14]. Dimitrou et al. reported that insensible water loss was lower and urine output higher in preterms who were exposed to prenatal lung maturation [17]. The absence of significant differences in the current cohort can be explained by their overall high exposure rate (87%).

Obviously, our study has its limitations. The retrospective analysis of this cohort presents inherent limitations in establishing causation. Future research should aim to unveil normative values and optimize sodium and fluid administration protocols. Additionally, the association of different variables with sodium homeostasis in ELBW infants should be established in additional longitudinal assessments. While we have explored the impact of SGA or AGA on sodium serum trends, we have not explored potential differences in sodium handling in later life [18]. Because of the retrospective design, we were unable to capture potential osmotic diuresis, associated with hyperglycemia, and the fluid regimen was somewhat different from the latest guidelines [19,20].

To conclude, we confirmed sodium serum patterns and reference values in ELBW cases over postnatal age (day 1–10), while additional variables of these sodium trends were suggested. While associations between sodium serum levels and birth weight, gestational age, or length at birth are in line with what has been previously described in the literature, we observed the association between exposure to inotropic agents or ibuprofen, and serum sodium values, in ELBW infants. This should be further prospectively validated and confirmed in larger cohorts. Such reference values are useful to provide clinicians guidance to interpret single sodium observations or postnatal trends in individual ELBW newborns.

## Figures and Tables

**Table 1:** Demographic variables of the extremely low-birth-weight (ELBW) cohort (AGA: appropriate for gestational age; SGA: small for gestational age).

Variable		Statistic	All
**Birth weight (g)**		N	211
		Mean	807
		Std	135
		Median	830
		IQR	(715; 910)
		Range	(370; 1000)
**Length (cm)**		N	200
		Mean	34
		Std	2.4
		Median	34
		IQR	(32; 36)
		Range	(28; 40)
**Growth category**	AGA	n/N (%)	170/211 (80.6%)
	SGA	n/N (%)	41/211 (19.4%)
**Gestational age (weeks)**		N	211
		Mean	26.7
		Std	1.9
		Median	26
		IQR	(25; 28)
		Range	(23; 34)
**Sex**	Female	n/N (%)	99/211 (46.9%)
	Male	n/N (%)	111/211 (52.6%)
	Unknown	n/N (%)	1/211 (0.5%)
**Neonatal death**	No	n/N (%)	183/211 (86.7%)
	Yes	n/N (%)	28/211 (13.3%)
**Lung maturation**	No	n/N (%)	28/207 (13.5%)
	Yes	n/N (%)	179/207 (86.5%)
**Caesarean**	No	n/N (%)	63/209 (30.1%)
	Yes	n/N (%)	146/209 (69.9%)
**Ibuprofen**	No	n/N (%)	79/209 (37.8%)
	Yes	n/N (%)	130/209 (62.2%)
**Inotropic agents**	No	n/N (%)	111/208 (53.4%)
	Yes	n/N (%)	97/208 (46.6%)

**Table 2:** Mean (95% CI) daily and mean difference compared to day 1 (95% CI) in serum sodium concentrations in extremely low-birth-weight (ELBW) infants for the consecutive subsequent days (day 2–day 10, with 95% confidence intervals (CI) in the cohort.

Day	Mean Estimate (95% CI)	Mean Difference vs. Day 1 (95% CI)	*p*-Value
Day 1	138.7 (138.3; 139.1)	0	.
Day 2	144.4 (143.7; 145.0)	5.7 (5.0; 6.3)	<0.0001
Day 3	145.4 (144.8; 146.0)	6.7 (6.0; 7.5)	<0.0001
Day 4	143.7 (143.1; 144.4)	5.0 (4.3; 5.8)	<0.0001
Day 5	142.6 (141.9; 143.4)	3.9 (3.1; 4.8)	<0.0001
Day 6	141.6 (140.9; 142.2)	2.9 (2.0; 3.7)	<0.0001
Day 7	140.3 (139.5; 141.1)	1.6 (0.7; 2.5)	0.0005
Day 8	139.8 (139.0; 140.6)	1.1 (0.2; 2.0)	0.0141
Day 9	139.7 (138.8; 140.5)	1.0 (0.0; 1.9)	0.0422
Day 10	140.3 (139.4; 141.2)	1.6 (0.6; 2.6)	0.0015

## Data Availability

The original contributions presented in this study are included in the article and Supplementary Materials. Further inquiries can be directed to the corresponding author.

## Supplementary Materials

The following supporting information can be downloaded at https://www.mdpi.com/article/10.3390/children12030337/s1, Table S1: Effect of caesarean on serum sodium concentration of ELBW infants in the first 10 days of life; Table S2: Effect of gestational age on serum sodium concentrations of ELBW infants in the first 10 days of life; Table S3: Effect of birth weight on serum sodium concentrations of ELBW infants in the first 10 days of life; Table S4: A comparison of serum sodium levels during the first 10 days in ELBW infants who were exposed to ibuprofen (yes/no) versus those who were not, with a 95% confidence interval; Table S5: A comparison of serum sodium levels during the first 10 days in ELBW infants who were exposed to inotropic agents (yes/no) versus those who were not, with a 95% confidence interval; and Table S6: The interaction between gestational age and birth weight with effect on serum sodium values in the first 10-days of life, with a 95% confidence interval.
