# Supplementary material for: Sodium Patterns and Their Variables in a Cohort of ELBW Infants in the First 10 Days of Life
Source: Children (Basel). 2025 Mar 7;12(3):337. doi: 10.3390/children12030337 (PMC11941669; doi:10.3390/children12030337)
Supplement: Supplementary file 1 [file children-12-00337-s001.zip › children-3519029-supplementary.pdf]

Supplemental materials

Table S1. Effect of caesarean on serum sodium concentration of ELBW infants in the first 10 days of life.

Table S2. Effect of gestational age on serum sodium concentrations of ELBW infants in the first 10 days of life.

Table S3. Effect of birth weight on serum sodium concentrations of ELBW infants in the first 10 days of life.

Table S4. A comparison of serum sodium levels during the first 10 days in ELBW infants who were exposed to ibuprofen (yes/no) versus those who were not, with a 95% confidence interval.

Table S5. A comparison of serum sodium levels during the first 10 days in ELBW infants who were exposed to inotropic agents (yes/no) versus those who were not, with a 95% confidence interval.

Table S6. The interaction between gestational age and birth weight with effect on serum sodium values in the first 10-days of life, with a 95% confidence interval.

Table S1: Effect of caesarean delivery on serum sodium concentration of ELBW infants in the first 10 days of life from in the University Hospitals Leuven cohort.

	**Mean estimate (95% CI)**			
**Day**	**No caesarean**	**Caesarean**	**Mean difference (95% CI)**	**P-value**
Day 1	138.1 (137.4;138.9)	139.0 (138.4;139.5)	-0.8 (-1.8;0.1)	0.0925
Day 2	145.2 (144.1;146.3)	144.0 (143.3;144.7)	1.2 (-0.1;2.5)	0.0758
Day 3	146.5 (145.4;147.6)	144.9 (144.2;145.6)	1.6 (0.3;2.9)	0.0156
Day 4	144.8 (143.7;146.0)	143.2 (142.5;144.0)	1.6 (0.3;3.0)	0.0196
Day 5	143.3 (142.0;144.7)	142.3 (141.4;143.2)	1.0 (-0.6;2.6)	0.2373
Day 6	142.4 (141.1;143.6)	141.2 (140.4;142.0)	1.1 (-0.3;2.6)	0.1259
Day 7	140.7 (139.3;142.1)	140.3 (139.3;141.3)	0.4 (-1.3;2.1)	0.6266
Day 8	140.8 (139.4;142.2)	139.5 (138.5;140.4)	1.3 (-0.4;3.0)	0.1312
Day 9	140.6 (139.1;142.0)	139.3 (138.3;140.3)	1.2 (-0.5;3.0)	0.1692
Day 10	142.1 (140.5;143.6)	139.6 (138.5;140.6)	2.5 (0.6;4.4)	0.0101
CI: confidence interval<br>P-value Day × group interaction: 0.0222				

Table S2: Effect of gestational age on serum sodium concentrations of ELBW infants in the first 10 days of life in the University Hospitals Leuven cohort.

Day	Slope (95% CI)	P-value
1	0.24 (0.01;0.47)	0.0432
2	-0.32 (-0.65;0.02)	0.0667
3	-0.78 (-1.09;-0.47)	<0.0001
4	-0.78 (-1.10;-0.46)	<0.0001
5	-0.88 (-1.27;-0.49)	<0.0001
6	-0.79 (-1.15;-0.44)	<0.0001
7	-0.91 (-1.33;-0.48)	<0.0001
8	-0.87 (-1.28;-0.46)	<0.0001
9	-0.89 (-1.34;-0.44)	0.0001
10	-1.20 (-1.66;-0.73)	<0.0001

CI: confidence interval
Slope = change in sodium for a 1-week higher gestational age
P-value Day × predictor interaction: <0.0001

Table S3: Effect of birth weight on serum sodium concentrations of ELBW infants in the first 10 days of life in the University Hospitals Leuven cohort.

Day	Slope (95% CI)	P-value
1	-0.99 (-2.65;0.68)	0.2428
2	-1.76 (-4.13;0.60)	0.1435
3	-3.64 (-5.92;-1.37)	0.0018
4	-4.28 (-6.60;-1.96)	0.0004
5	-3.85 (-6.60;-1.11)	0.0061
6	-5.19 (-7.69;-2.69)	<0.0001
7	-8.08 (-10.82;-5.33)	<0.0001
8	-6.66 (-9.48;-3.84)	<0.0001
9	-7.24 (-10.23;-4.25)	<0.0001
10	-7.19 (-10.43;-3.94)	<0.0001

CI: confidence interval
Slope = change in sodium for a 2-fold higher birth weight
P-value Day × predictor interaction: 0.0019

Table S4: A comparison of serum sodium levels during the first 10 days in ELBW infants who were exposed ibuprofen (no/yes) versus those who were not, with a 95% confidence interval in the University Hospitals Leuven cohort.

	**Mean estimate (95% CI)**			
**Day**	**No**	**Yes**	**Mean difference (95% CI)**	**P-value**
Day 1	139.0 (138.3;139.7)	138.5 (137.9;139.0)	0.6 (-0.3;1.5)	0.2164
Day 2	143.7 (142.7;144.7)	144.7 (144.0;145.5)	-1.0 (-2.3;0.2)	0.1008
Day 3	145.1 (144.1;146.1)	145.6 (144.8;146.4)	-0.5 (-1.8;0.8)	0.4384
Day 4	143.5 (142.4;144.5)	143.9 (143.1;144.7)	-0.4 (-1.7;0.9)	0.5119
Day 5	142.0 (140.7;143.2)	143.0 (142.1;144.0)	-1.1 (-2.6;0.5)	0.1796
Day 6	140.1 (138.9;141.2)	142.4 (141.6;143.2)	-2.3 (-3.7;-1.0)	0.0010
Day 7	138.3 (137.0;139.6)	141.5 (140.5;142.4)	-3.2 (-4.8;-1.6)	0.0001
Day 8	137.7 (136.4;139.0)	141.1 (140.1;142.0)	-3.4 (-4.9;-1.8)	<0.0001
Day 9	136.9 (135.6;138.3)	141.1 (140.1;142.1)	-4.2 (-5.9;-2.5)	<0.0001
Day 10	137.8 (136.3;139.3)	141.6 (140.6;142.7)	-3.9 (-5.7;-2.1)	<0.0001

CI: confidence interval
P-value Day × group interaction: 0.0001

Table S5: A comparison of serum sodium levels during the initial 10 days in ELBW infants who were exposed to inotropic agents (no/yes) versus those who were not, with a 95% confidence interval in the University Hospitals Leuven cohort.

	**Mean estimate (95% CI)**			
**Day**	**No**	**Yes**	**Mean difference (95% CI)**	**P-value**
Day 1	138.9 (138.3;139.5)	138.4 (137.8;139.0)	0.5 (-0.4;1.3)	0.2866
Day 2	144.9 (144.0;145.7)	143.8 (142.9;144.7)	1.1 (-0.1;2.4)	0.0741
Day 3	145.5 (144.6;146.3)	145.3 (144.4;146.2)	0.2 (-1.1;1.4)	0.7938
Day 4	143.5 (142.7;144.4)	144.0 (143.0;144.9)	-0.4 (-1.7;0.8)	0.5115
Day 5	142.0 (141.0;143.1)	143.4 (142.3;144.5)	-1.3 (-2.8;0.2)	0.0833
Day 6	140.5 (139.6;141.4)	142.8 (141.9;143.8)	-2.4 (-3.7;-1.0)	0.0006
Day 7	138.7 (137.7;139.8)	142.1 (141.0;143.2)	-3.4 (-4.9;-1.9)	<0.0001
Day 8	138.2 (137.2;139.2)	141.8 (140.7;142.9)	-3.6 (-5.1;-2.1)	<0.0001
Day 9	138.5 (137.4;139.6)	141.2 (140.0;142.4)	-2.7 (-4.3;-1.1)	0.0013
Day 10	138.6 (137.4;139.8)	142.1 (140.8;143.3)	-3.5 (-5.2;-1.8)	<0.0001
CI: confidence interval<br>P-value Day × group interaction: <0.0001				

Table S6: The interaction between gestational age and birth weight with effect on serum sodium values in the first 10-days of life, with a 95% confidence interval.

**Test**	**P-value**
Gestational age by Birth weight interaction	
Effect of Birth weight given gestational age	<0.0001
Effect of Gestational age given birth weight	<0.0001

	**Gestational age**		**Birth weight**	
**Day**	**Slope (95% CI)**	**P-value**	**Slope (95% CI)**	**P-value**
**1**	0.33 (0.08;0.57)	0.0088	-1.82 (-3.56;-0.07)	0.0413
**2**	-0.26 (-0.62;0.10)	0.1576	-1.13 (-3.64;1.38)	0.3765
**3**	-0.69 (-1.02;-0.36)	<0.0001	-1.91 (-4.25;0.44)	0.1100
**4**	-0.65 (-0.98;-0.31)	0.0002	-2.67 (-5.07;-0.26)	0.0297
**5**	-0.78 (-1.20;-0.37)	0.0002	-1.92 (-4.76;0.92)	0.1841
**6**	-0.61 (-0.98;-0.24)	0.0014	-3.64 (-6.25;-1.03)	0.0066
**7**	-0.55 (-0.99;-0.12)	0.0121	-6.63 (-9.51;-3.76)	<0.0001
**8**	-0.62 (-1.04;-0.20)	0.0042	-5.12 (-8.04;-2.20)	0.0007
**9**	-0.60 (-1.07;-0.13)	0.0123	-5.83 (-8.99;-2.68)	0.0003
**10**	-0.95 (-1.43;-0.47)	0.0001	-5.19 (-8.46;-1.92)	0.0020

CI: confidence interval
Slope = change in sodium for a 1 week higher gestational age / 2-fold higher birth weight
P-value Day × Gestational age interaction: <0.0001
P-value Day × Birth weight interaction: 0.0337
